# A Nutrition Education Intervention Positively Affects the Diet–Health-Related Practices and Nutritional Status of Mothers and Children in a Pulse-Growing Community in Halaba, South Ethiopia †

**DOI:** 10.3390/children11111400

**Published:** 2024-11-19

**Authors:** Getahun Ersino Lombamo, Carol J. Henry, Gordon A. Zello

**Affiliations:** 1 Health Sciences Office, University of Saskatchewan, Saskatoon, SK S7N 5E5, Canada; 2 Division of Nutrition and Dietetics, College of Pharmacy and Nutrition, University of Saskatchewan, Saskatoon, SK S7N 5E5, Canada; carol.henry@usask.ca (C.J.H.); gordon.zello@usask.ca (G.A.Z.); 3 School of Nutrition, Food Science and Technology, Hawassa University, Hawassa P.O. Box 05, Ethiopia

**Keywords:** nutrition education, maternal nutrition, child nutrition, pulse, dietary intake, Ethiopia

## Abstract

Objective: We conducted a six-month nutrition education intervention focused on the consumption of pulses and other foods to assess the effect on knowledge, attitude and practice (KAP) as well as the nutritional status of children and mothers from two pulse-growing communities in Halaba, south Ethiopia. Methods: About 200 mother–child pairs in each of two purposively selected communities participated in this intervention study. A six-month nutrition education programme, involving interactive monthly community meetings and home visits, was offered to one of the two communities and the other served as a control/comparison. This study incorporated the use of Health Belief Model constructs to assess the KAP/perceptions of mothers surrounding pulse and other food consumptions, as well as nutrition-related issues before and after the intervention. Objective measures included dietary diversity scores (DDSs), one-day weighed dietary intakes and nutritional status measures based on anthropometric information. Demographics and socioeconomic information were also collected at baseline and endline. Results: Significant improvements (*p* < 0.05) were found in the intervention group on the KAP and perceptions of pulse nutrition benefits among mothers, DDSs and pulse and animal source food consumption indexes for mothers and children and the mean body-mass-index-for-age Z-score and wasting among children. Conclusions: Community-based nutrition education interventions involving monthly interactive community meetings and home visits in pulse-growing communities from a resource-poor country like Ethiopia can be effective in improving mothers’ knowledge of pulse nutrition and consumption frequency, leading to improvements in the DDSs of children and mothers while decreasing child underweight and wasting.

## 1. Introduction

Linking nutrition education with agricultural interventions has been reported to hold potential in improving the nutrition and health of agriculture-based communities in emerging countries [1]. Agricultural interventions focusing on production, with no or limited integration to nutrition outcomes, did not impact the nutritional status of children [1,2]. As a result, the integration of behaviour-change communication tools, such as a community-based nutrition education with agricultural interventions, has been promoted to improve the effects on nutritional health.

The production of pulse crops is one area of agriculture that can be leveraged to improve the nutritional health of agricultural communities. Pulses are low-fat legumes with up to twice the protein of cereals and rich in minerals like iron and zinc [3,4,5,6]. Inclusion of pulses in the diet has benefits such as complementing cereal-based staple foods to improve protein quality and contributing to nutrition security (e.g., of protein, micronutrients) [5]. Due to their higher market value than cereals, pulses can also be a source of cash income for households while serving as natural fertilizers (via their nitrogen-fixing ability), thereby, contributing to a more sustainable environment.

In Ethiopia, pulses are grown both for food and cash income [7]. Pulses play an important role in the diet by complementing foods from cereal staples as regular consumption of animal source protein is unaffordable by the rural poor [8]. However, malnutrition among children and mothers in rural Ethiopia, where over 80% of the population resides, has remained high despite gradual progress in the last decade [9,10,11].

Our baseline studies, conducted as part of a pre-intervention study in Halaba (a pulse-growing district in south Ethiopia), showed a high prevalence of maternal and child undernutrition, limited consumption pulses, low dietary diversity and low dietary intakes [12,13,14]. Also, mothers from the pulse-growing communities perceived greater barriers to pulse consumption while scoring lower on the severity of child malnutrition (i.e., they did not think that child malnutrition was of such significant concern) compared with mothers from the cereal-growing communities. A cross-sectional study of adolescent girls in the same region reported high undernutrition but infrequent consumption of pulses resulting in limited contribution to the protein and iron intakes of the girls [15]. The study speculated that nutrition education focusing on potential barriers and benefits of pulses may improve the frequency of consumption and provide nutrition benefits.

The production of pulses could be geared toward improving diets; however, intervention studies focusing on the production and use of pulses to improve the nutritional status in Ethiopia are limited. Recent studies [16,17] have shown that nutrition education interventions can be effective approaches to promoting awareness, knowledge and skills in household pulse food preparation. Participatory pulse agriculture nutrition intervention research in rural communities of northern Malawi has shown improvement in child undernutrition [18].

The objective of the current study was to assess the effect of a nutrition education intervention focusing on pulse production and consumption on the knowledge, attitudes, practices and nutrition of mothers and children in two leading pulse-growing communities from rural Halaba, south Ethiopia. Based on our baseline studies, we designed and implemented a six-month educational nutrition intervention following the principles of the Health Belief Model. We emphasised the multiple benefits of pulses to tackle the perceived barriers to consumption.

## 2. Methods

### 2.1. Study Setting and Participants

This study was conducted in two rural communities in Halaba, known traditionally as a pulse- and pepper-producing region. The district (Woreda) studied is in the southern part of Ethiopia and administratively affiliated to the Southern Nations, Nationalities and People’s Region (SNNPR). Two rural communities that were identified by the local agriculture office as leading pulse producers were selected for this study. The selection of the study communities was purposive in alignment with the objective of the intervention, which focused on the production and utilization of pulse crops and the effect on nutrition of mothers and children in these communities. The local names of the two communities are Guba-Sherero and Holagoba-Kukie.

We targeted mothers with their youngest < 5 years of age child. In each community, about 200 mother–child pairs were randomly selected. The sample size was initially determined for a larger baseline study [12,14] using a formula for cross-sectional studies [19]. Eligible households for this study were those with a mother and < 5 years of age child.

Following our baseline study [12,14], the two communities were assigned as control and intervention in a quasi-experimental design. Holagoba-Kukie, which had a higher prevalence of child stunting and maternal undernutrition, was purposely assigned to receive the intervention, and Guba-Sherero served as the control or comparison community. The two communities, although in the same district, were far enough apart to prevent potential contamination of the education intervention to the unintended community.

Ethics clearance was obtained from the Behavioural Ethics Board (BEH#12-357) of the University of Saskatchewan, as well as the Regional Health Bureau of SNNPR. All mothers gave oral consent after the purpose of the research was read and explained to them by the local research assistants. This study was conducted between March 2013 and April 2014.

### 2.2. Intervention

A six-month educational nutrition intervention was given to participants in Holagoba-Kukie following the baseline study. The baseline results [12,14] were used to inform the education intervention (i.e., in selecting the intervention-community, topic/contents, delivery strategies). The use of theories to guide nutrition interventions was shown to be more effective in promoting certain health behaviour than those with no theoretical foundation [20,21]. The Health Belief Model (HBM) is one of the earliest and widely used theoretical models of behaviour. The main premise of the model is that people’s *perception* of the threat posed by a certain health problem or behaviour, and *benefits* of and *barrier* to proposed behaviour/action, determines their readiness to change toward the desired health behaviour or action [20,21]. The model has six constructs (i.e., perceived *susceptibility*, *severity*, *benefits*, *barriers*, *cues* for action and *self-efficacy*). Part of the baseline study [22] assessed mothers’ perceptions around susceptibility to malnutrition, severity of poor dietary practices and benefits and barriers to pulse consumption by following the constructs of the HBM (see Section 2.3 for more on this). For example, one of the pre-intervention findings was that mothers perceived a greater barrier to and lower scores on benefits of pulse consumption. Such findings helped to inform the focus/topic and content of the educational intervention. The education generally aimed at promoting pulses and pulse-based foods as part of healthy meals.

The intervention targeted mothers participating in our study; however, husbands, others residing in the household and community members were also encouraged to participate to create a supportive environment for the mothers.

The education was facilitated by two nutrition educators (a nutrition graduate and a nurse) along with two health-extension and one agriculture-extension workers who spoke the local language. The two nutrition educators received four days of training, by GE, focusing on data collection and the delivery of the intervention. The nutrition educators were part of the baseline study and already accustomed to the communities. The health- and agriculture-extension workers supported the intervention by reinforcing the messages during their routine contact with the local mothers and farmers. Supervision at the field level was carried out by GE.

The intervention had six monthly group sessions, as well as 1–2 monthly home visits to participant households. Mothers came to a central location (i.e., Health Post) at the beginning of each month to learn and converse on the topic for that month. Following each monthly session, educators visited each household to further discuss the topic and respond to any questions the mothers had. At the beginning of each monthly session, topics covered previously were reviewed and questions discussed together.

The education programme contained six basic nutrition-related topics for each month, framed based on previous studies and our baseline findings. A summary of the topics, content highlights and key nutrition messages are presented in the Supplementary Table S1. To aid the discussion of the six topics, a large poster was prepared, and educators were given a laminated copy of mini posters. Educators were also given a small booklet prepared for the intervention to help in facilitating group sessions. The booklet contained a brief introduction on each topic, interactive learning activities and key nutrition messages.

### 2.3. Data Collection

A structured questionnaire was used to interview the mothers from both communities. The questionnaire asked for participants’ demographic and socioeconomic status, and mothers’ knowledge of nutrition/pulse, attitudes and practices. Items that assessed dietary diversity and consumption frequency of certain foods were also included. For easy comparison of socioeconomic status between communities, a wealth index was developed using various household-owned assets (oxen, donkey/horse cart, bicycle, motorcycle, radio, television, mobile phone and hand torches), housing characteristics (types of roofing, flooring, windows and number of rooms) and presence or absence of an improved sanitation facility. This approach has been described in detail in our earlier work [12]. Characterising socioeconomic status using an asset-based approach is common in low-income countries where most households do not have a regular monthly income [23]. For the dietary diversity, we adapted the FAO questionnaire to reflect local foods [24]. The questionnaire listed 16 food groups (i.e., cereals; white tubers and roots; vitamin A–rich vegetables and tubers; dark green leafy vegetables; other vegetables; vitamin A–rich fruits; other fruits; organ meat; flesh meats; eggs; fish and seafood; legumes, nuts and seeds; milk and milk products; oils and fats; sweets; and spices, condiments, beverages).

The questionnaire also included 18 nutrition- and pulse-related questions for the mothers based on the six constructs of the Health Belief Model [25]—three items for each of the six-constructs (i.e., perceived susceptibility and severity, perceived benefits and barriers as well as self-efficacy and cues for action). Each of 18 items were asked on a 5-point Likert scale (1 = strongly disagree to 5 = strongly agree). The questions asked mothers’ perception of their (and their children’s) susceptibility to and severity of consequences of poor dietary practices, mothers’ perceptions of benefits and barriers to pulse consumption and cues for consumption of pulses and self-efficacy to take steps to healthier dietary practices.

In addition to the questionnaire, one-day in-house weighed food records were collected in a subsample of the study participants using portable weighing scales (Model CS 2000, Ohaus Corporation, Parsippany, NJ, USA) in both the intervention (59 mothers and 59 children) and control (63 mothers and 63 children) communities. All questionnaire-based data and weighed food records were collected by trained female data collectors, supervised by GE and nutrition research assistants. Interviews were carried out at the residences of participants or at the nearest community centre (schools, health centres or health posts). Nutrient values (for energy, protein, iron, zinc and calcium) per 100 g of edible food were obtained from Ethiopian food composition tables [26,27]. Food samples that did not have nutrient values in the food composition tables were analysed according to approved AACC international methods as described in our previous work [28].

Following standard procedure and tools [29], anthropometric measurements (height in centimetres, weight in kilograms and mid-upper arm circumference in centimetres) were collected from mother–child pairs at baseline and endline. To minimize measurer error, GE carried out all anthropometric measurements.

### 2.4. Statistical Analyses

All questionnaire-based data were entered into SPSS spreadsheets (SPSS Statistics Version 20, IBM Corp., Armonk, NY, USA). Background information such as wealth index, household size and amount of pulse harvest were presented as a median with a interquartile range (IQR). Median values were chosen over mean for certain variables due to non-normal distribution. The KAP-related items were expressed as percentages. The 18 items based on the six HBM domains were summarized as average scores (average of three items per domain) for each mother. Then, the group average for each of the six domains was calculated for intervention and control community at baseline and endline. Dietary diversity data were analysed by aggregating the 16 food groups into 9 groups, according to the FAO guide [24]. A DDS was calculated for each mother and child by assigning a score of “1” or “0” depending on whether they had eaten from each of the nine main food groups. We have reported median DDSs with IQR. A consumption index was calculated for selected food groups (e.g., pulses, animal source foods), based on the frequency of consumption, which was then expressed as the median and IQR. A consumption index of 1 = least frequent (≤2 per month), 2 = 1–2 times per week, 3 = 3–6 times per week and 4 = most frequent (≥1 per day). Dietary intakes of the selected nutrients were expressed as the median and the percent of recommended nutrient intake (RNI). Mothers’ weight and height measurements were converted to body mass index (BMI), expressed as kilogram of body weight divided by the square of height in metres (kg/m^2^). Mothers’ BMI was then classified into underweight (BMI < 18.5), normal (18.5 < BIM < 24.9) and overweight (BMI > 24.9). The WHO Anthro (ver. 3.2.2, 2011) was used to convert the children’s anthropometric measurements into height-for-age, weight-for-height, weight-for-age and BMI-for-age Z-scores before transferring to SPSS for further analysis. The paired samples *t*-test were used for comparing means and the McNemar test for prevalence estimates between baseline and endline within the two communities. The independent sample *t*-test for means and the chi-squared test for prevalence estimates were employed in comparing the two groups. The Mann–Whitney *U* and Wilcoxon’s tests were also used for the nonparametric data to determine between and within group differences, respectively. Statistical significance was set at *p* < 0.05.

## 3. Results

Results have been summarized for mother–child pairs in the control (*n* = 180) and intervention (*n* = 183) communities that completed the baseline and endline study. Individual and household characteristics of the communities are presented in Table 1. Median household size, maternal age and wealth index, as well as mothers and their husbands’ formal education status, were similar between the control and intervention communities. The size of land and pulses crops produced in the recent harvest preceding this study appeared to be slightly better for the control/comparison than the intervention community (*p* < 0.001). Almost all households (90 to 100%) in either community identified themselves as pulse growers.

### 3.1. Knowledge, Attitude and Practice (KAP)

There were no significant differences in the reported “knowledge of balanced diets” and knowledge of “nutritional benefits of pulses” at baseline between the groups (Table 2). The knowledge of these two items improved significantly both within and between groups following the intervention (*p* < 0.01), and the percent change in improvement was twice to four times greater in the intervention than the control group.

Use of pulses for home consumption and market improved in both groups following the intervention (*p* < 0.01), and improvement was better in the intervention group. Reported consumption of pulses or pulse-based foods by mothers and their children was already high at baseline in both groups, particularly in the intervention (*p* < 0.01). Following the intervention, responses to this item remained high in the intervention group while it also significantly improved in the control group (*p* < 0.01). Mothers’ positive attitude toward pulse-based foods was already higher at baseline in the intervention group (*p* < 0.01) and remained even higher following the intervention (*p* < 0.01). Within group improvements were also significant (*p* < 0.01) and of similar magnitude in both groups following the intervention.

### 3.2. Scores on the Constructs of the HBM

Average scores on perceived susceptibility and severity to consequences of poor dietary practices, scores on perceived benefits of pulses and barriers to their consumption, as well as scores on cues for consumption of pulses and self-efficacy (of mothers to take steps to healthier dietary practices) significantly improved following the intervention in both groups, but improvements were significantly higher (*p* < 0.001) for the intervention compared with the control group (Table 3).

The perceived benefit of pulses did not significantly improve in the control community while it did in the intervention group (*p* < 0.001). While the average scores for perceived barriers to consumption of pulses were not significantly different at baseline, they dropped significantly in both groups following the intervention, but the drop was almost twice as high in the intervention as it was in the control group (*p* < 0.001).

### 3.3. Frequency of Consumption Index and Selected Food Groups and Diet Diversity

Table 4A presents mothers and children’s frequency of consumption from certain food groups, summarized as consumption indexes, as well as median dietary diversity scores with 25th–75th percentile values. Consumption index of mothers for animal source foods did not differ at baseline between groups, but it significantly improved in the intervention group following the intervention (*p* < 0.05) while it remained unchanged in the control group. The consumption index of fruit and vegetable for mothers was slightly different at baseline between groups but did not differ within or between groups following the intervention. The pulse consumption index of mothers was better for the intervention community at baseline compared with the control-community (*p* < 0.001); at endline, mothers in both groups improved their consumption index of pulses (*p* < 0.001) but there is no significant difference between groups.

The diet diversity of mothers in the intervention community was significantly lower at baseline compared with the control (*p* < 0.05), but it significantly improved in the intervention group at endline while it significantly dropped for the control community (*p* < 0.05).

Similarly, consumption index of animal source foods for children was similar for both groups at baseline but significantly improved in the intervention group (*p* < 0.05) while it remained unchanged in the control community. The fruit and vegetable consumption index for the children was also already better at baseline for the intervention group (*p* < 0.001) and remained high at endline but did not significantly differ between or within groups. The pulse consumption index was significantly better at baseline for the intervention group (*p* < 0.001), and the index improved significantly within groups following the intervention (*p* < 0.05).

The median DDS for the children were similar at baseline but both communities had improved diet diversity, although it was not significant between groups following the intervention.

### 3.4. Average Intakes of Energy and Selected Nutrients (In a Subsample)

The median energy intake of mothers was slightly above 50% of the RNI (Table 4B). There was no significant difference between or within groups following the intervention. However, mothers in the intervention community had slightly higher median values for energy compared with those in the control at baseline (*p* < 005). Median intakes of protein for the mothers were above the RNI both at baseline and endline and did not significantly differ between or within groups after the intervention. Median protein intake at baseline was higher for the intervention than the control group (*p* < 0.05).

Iron intakes of mothers, which were similar in both groups at baseline, improved significantly in the control (*p* < 0.01) as well as in the intervention (*p* < 0.05) groups at endline, but these did not differ between groups. Zinc intakes did not change significantly at baseline or endline in the within or between group design. Calcium, although similar at baseline, dropped significantly for the intervention group both in the within and between group design (*p* < 0.05).

Energy, protein, zinc and calcium intakes for the children were similar at baseline but increased significantly in both groups following the intervention (*p* < 0.001) and endline values were not significantly different between child groups. Iron intakes increased significantly in both groups following the intervention (*p* < 0.001) and were also significantly different at baseline (*p* < 0.05).

### 3.5. Maternal and Child Anthropometry

There were no significant differences between groups in mean height, MUAC, weight and BMI of mothers both at baseline and endline in either group (Table 5). Levels of maternal undernutrition (% BMI < 18 kg/m^2^) slightly dropped after the intervention in the control community but remained the same in the intervention group. Differences were not significant between or within groups in either time point.

The mean HAZ of the children significantly dropped (worsened) in both communities (*p* < 0.05), but the magnitude of the drop was greater in the control (−0.37) than the intervention (−0.29) community. The mean BMI-for-age and rate of wasting among children significantly improved in both communities (*p* < 0.05).

Similarly, the prevalence of stunting significantly increased in both the control and the intervention community following the six-month intervention, and it was worse in the intervention group both at baseline and endline (*p* < 0.05). In contrast, prevalence of wasting, although not different between groups in either time point, declined significantly in both groups following the intervention (*p* < 0.05). Prevalence of underweight remained unchanged within groups, although it appeared to drop slightly in the intervention community.

## 4. Discussion

Following the six-month nutrition education intervention, our findings indicated mothers’ knowledge of balanced diets, and the nutritional benefits of pulses significantly improved in the intervention community. Reported consumption, and intentions to consume pulse or pulse-based foods, as well as positive attitudes toward pulse foods have improved significantly in both groups. The consumption index for animal source foods improved for mother–children in the intervention group after the six-month education, but no change was observed in the control community. Median dietary diversity scores (DDSs) dropped for control mothers, but significantly improved for those in the intervention community. Median DDSs of intervention children also improved following the education; although the overall DDS improved for control children, the median value remained unchanged.

The nutrition education we implemented in this study was basic and tailored to the specific community (i.e., a pulse-growing rural community with low nutrition literacy). In addition to educating the community on key nutrition specific issues, the intervention emphasized nutritional benefits of incorporating pulses in family meals, and other benefits of pulses. Positive or “gain-framed” messaging, as opposed negative or “loss-framed” messaging, has been indicated to be effective, particularly in individuals/communities with limited knowledge of the subject matter [33]. Negative messaging of health education has also been shown to be effective in bringing behaviour change, but only among the educated populous and if the negative message was strong enough to be perceived as of greater threat [34]. However, in the current study, we encouraged only positive messaging, such as the benefits of consuming pulses and a variety of foods from different food groups for the mother’s and children’s health. This was particularly important as the principal targets of the intervention were women, the majority of whom had no formal schooling (Table 1).

Consensus exists that the old expert-driven approach to nutrition education is ineffective as it focuses on information transfer from experts to an audience [35]. Instead, participatory approaches involving the target population in the planning, implementation and evaluation processes have been suggested more effective in achieving desired change, with an increased chance of sustainability [36]. In the current intervention study, we applied a number of principles of participatory research as follows: conducting a formative research to understand nutrition-related concerns specific to the study communities, a selection of nutrition topics and content based on the formative research, the use of locally trained people to lead the education intervention, allowing experience sharing and discussions by local mothers during the monthly sessions and home visits and involving other household members (husbands, extended family members) and neighbours. Community-based education programmes in northern Malawi involving various household members, such as grandmothers, and discussing multiple issues (such as gender, feeding practices) have showed positive effects on child feeding practices [37,38].

Dietary diversity scores have been used as proxy measures of both household’s socio-economic ability to access certain foods and the quality and micronutrient adequacy of diets consumed by individuals such as women and children [24,39]. Our baseline and similar other studies in developing countries have shown an association between the maternal and child DDS [13,14,40]; DDSs have also been associated with maternal undernutrition and child stunting [41,42]. The lack of adequate dietary diversity among various subsistence farming household members, particularly children, has been reported by several studies in Ethiopia [28,43,44,45,46,47,48]. Our six-month educational intervention significantly improved dietary diversity in both mothers and children of the intervention community; the increment of the DDS in the intervention community was mainly due to the increased proportion of participants who reported consuming pulse-based foods during the second 24-hour dietary diversity recall. This improvement can be attributed to the intervention. The significant increase in pulse-consumption indexes in this group also supports the positive effect of the intervention in increasing the consumption frequency of pulse foods. However, the actual DDS of mothers and children only met the minimum requirements for DDSs at best. Strategies such as increasing nutrition literacy around food groups, the growing of diverse vegetables and fruits in addition to pulses, increasing women’s access to land and reducing women’s work burden should be part of future nutrition intervention efforts to move the median dietary diversity scores above the cut-points (i.e., a DDS of 4 to 5) for minimum requirements [49].

Findings based on the *constructs* of HBM (for mothers) also showed improvements in both communities, but greater improvements on the scores were observed for the intervention community. In particular, “perceived benefits of pulses” improved significantly only in the intervention community, and scores on “perceived barriers to consumption of pulses” also dropped nearly twice as much in the intervention compared with the control community. Community-based intervention studies that applied the HBM constructs in rural communities of south Ethiopia have also reported improvement in knowledge, attitude and practice in areas of child feeding, as well as improvement in wasting and underweight, but not stunting, in the intervention communities [16,50]. This study and similar ones suggest that using the Health Belief Model (HBM) can improve nutrition education effectiveness by helping to identify perceived threats, benefits and barriers to healthy behaviours. Indeed, improvements in maternal anthropometry such as mean BMI or the proportion with a low BMI of <18.5 kgm^−2^ did not differ significantly in either group in the pre-test–post-test design (Table 5). This may be due to the lack of impact on gender and other socioeconomic and demographic factors (such as mothers’ work burden, lack of access to resources, empowerment imbalance and physiological density) described in our earlier studies [12,14] which were not the focus of the current intervention study. Additionally, the intervention might not have been long enough or/and specific enough to impact levels of maternal undernutrition.

We reported significant improvements in child anthropometric outcomes, such as mean BMI-for-age Z-scores and prevalence of child wasting for the intervention group; however, similar improvement was observed in the control/comparison community as well, hence further investigation might be needed to account for all confounding factors. A positive effect on short-term nutrition status markers of anthropometric indices has also been reported in similar communities elsewhere after a six-month educational intervention [16]. Long-term nutritional status markers of anthropometric indices (such as height-for-age Z-score, stunting and underweight prevalence) did not improve or became worse after six months in this study—although the magnitude of the drop in mean HAZ score in the intervention group was not as big as in the control. The lack of or only limited improvements in the long-term nutritional status markers after a six-month intervention has also been reported by Mulualem et al. [16] and another recipe-based intervention [17] in similar settings elsewhere in Ethiopia. This may indicate that the effect of educational nutrition interventions on chronic undernutrition indicators may not be fully captured, or the interventions may not have been specific and/or long enough to impact these indices.

Dietary intakes of energy and the selected nutrients among intervention mothers did not show significant improvement, or the improvement did not differ from those in the control group; however, % RNI values of median intake remained above the RNI, except for energy and calcium. Dietary intakes of children, however, significantly improved in both the control and treatment groups (Table 4B). This was not unexpected as the children were, at least, six months older than what they were at baseline and expected to have an increased dietary intake. However, improvement in the median values of the energy intake and selected nutrients did not differ between groups after the intervention except for iron, which was also significantly different between the groups at baseline. A study among young children in a similar setting by Negash et al. [17] also reported a lack of significant changes in energy, iron and other macronutrient intakes following a six-month nutrition education. Again, this may indicate a lack of specificity of the intervention to impact the quantity of dietary intake, although the increased DDS meant improvement in the overall diet quality. Other factors (such as food supply and access to food), which we did not control for in the current design, might have also contributed.

This study was among the first few intervention control studies, if not the only, that attempted to comprehensively assess the effect of a six-month educational nutrition intervention on the perceptions of benefits, knowledge, attitude and practice (including consumption frequency of certain foods), as well as on dietary intake and nutritional status of mothers and children in pulse growing Ethiopian agricultural communities. The intervention was also carried out mostly by utilizing existing infrastructure (the government-run health and agriculture extension programmes), mobilizing local people and keeping expert involvement to the minimum, thus increasing its feasibility for wider application in other settings with minimal budget implications. However, this study did not detect a significant positive impact on long-term markers of child undernutrition, such as the mean HAZ score and stunting and underweight prevalence for the reason we speculated earlier. The results may be generalizable to similar pulse-growing communities but the generalizability of the findings to non-pulse-growing communities may be limited, and the education may need to be adapted to new settings.

## 5. Conclusions

Overall, our findings indicated that a community-based nutrition education intervention, involving monthly interactive community conversation meetings and home visits, in traditionally pulse-growing communities could be effective in improving knowledge on the benefits of pulses among rural mothers, pulse consumption frequency, and the subsequent improvement in dietary diversity among mothers and children in rural Ethiopia; as well, improvement in the prevalence of short-term markers of undernutrition such as mean BMI-for-age Z-scores and prevalence of wasting among children was found in the study communities. The application of the principles of the HBM theory also appeared to strengthen the design and implementation of the education, making it relevant for the specific community.

## Figures and Tables

**Table 1:** Baseline characteristics of participants and households from two pulse-producing communities in rural Halaba, south Ethiopia.

	Control	Intervention
	*n* = 180	*n* = 183
	Median (IQR)	Median (IQR)
Maternal age (y)	27 (25–30)	29 (25–32)
Household size	6 (4–7)	6 (4–7)
Wealth index	5 (3–8)	5 (3–7.6)
Size of cultivable land (ha)	1 (0.5–1.5)	0.75 (0.5–1) ^b^
Pulse produces from recent harvest (quintals) ^a^	3 (2–4)	2 (1.88–3.0) ^b^
	*n* (%)	*n* (%)
Number of children under five years of age		
One	79/180 (43.9)	127/183 (69.4)
Two or more	101/180 (56.1)	56/183 (30.6)
Mothers’ formal education		
No formal education	140/180 (77.8)	151/183 (82.5)
Primary or above	40/180 (22.2)	32/183 (17.5)
Husbands’ formal education		
No formal education	99/180 (55)	98/183 (53.6)
Primary level (1–6 grades)	58/180 (32.2)	55/183 (30.1)
Post primary (>6th grade)	23/180 (12.3)	30/183 (16.4)
Growing pulse crops		
Yes	161/180 (89.4)	183/183 (100)

IQR, interquartile range; ^a^ Estimate was based on *n* = 150 households in each community that declared that amount of pulse harvest; ^b^ Difference was significant at *p* < 0.001 (*Mann–Whitney U test*).

**Table 2:** Nutrition–pulse-related knowledge, attitudes and practice of mothers from two pulse-growing rural communities of Halaba, south Ethiopia.

	Frequency (%)			
	Control		Intervention	
	Pre (*n* = 180)	Post (*n* = 180)	Pre (*n* = 183)	Post (*n* = 183)
Mothers’ knowledge of balanced/varied diet	53/180 (29.4)	81/180 (45)	42/183 (23)	167/183 (91.3) ^bc^
Use of pulses both for home and market (income) ^a^	121/161 (75.2) ^c^	146/161 (90.7)	136/183 (74.3)	175/183 (95.6) ^cd^
Consumption of pulse/foods from pulse by mother–child	153/180 (85) ^bc^	180/180 (100)	181/183 (98.9)	183/183 (100)
Positive attitude toward pulse-based foods	131/180 (72.8) ^bc^	155/180 (86)	152/183 (83)	183/183 (100) ^bc^
Knowledge of nutritional benefit of pulses	82/180 (45.6) ^c^	131/180 (72.8)	69/183 (37.7)	181/183 (98.9) ^bc^
Intention to consume more pulse or pulse-based food in the future	109/180 (60.6) ^c^	148/180 (82.2)	140/183 (76.5)	181/183 (98.9) ^bc^

^a^ sample size of the control group for this variable is 161; ^b^ significant at *p* < 0.01 between respective groups *(chi-squared test)*; ^c^ significant at *p* < 0.01 within respective groups *(McNemar test);* ^d^ *p* = 0.051 between respective groups (*chi-squared test*).

**Table 3:** Average scores on the six main HBM domains for mothers from two pulse-growing rural communities of Halaba, southern Ethiopia (with a 5-point Likert scale).

	Mean ± Standard Deviation			
	Control		Intervention	
	Pre (*n* = 180)	Post (*n* = 180)	Pre (*n* = 183)	Post (*n* = 183)
Perceived susceptibility to consequences of poor dietary practices	3.41 ± 0.86 **	3.68 ± 0.55	3.68 ± 0.97	3.88 ± 0.48 ***
Perceived severity of consequences of Poor dietary practices	3.15 ± 0.81 **	3.59 ± 0.55	3.4 ± 0.86	3.79 ± 0.57 ***
Perceived benefits of pulses	3.42 ± 0.76 **	3.51 ± 0.62	3.63 ± 0.72	3.91 ± 0.54 ***
Perceived barriers to consumption of pulses	2.71 ± 0.95 ^a^	2.22 ± 0.62	2.72 ± 0.91	1.86 ± 0.51 ***
Cues for consumption of pulses	3.32 ± 0.84 **	3.59 ± 0.72	3.9 ± 0.74	4.14 ± 0.37 ***
Self-efficacy (of mothers to take healthy steps to dietary practices)	3.1 ± 0.91 **	3.71 ± 0.66	3.36 ±0.8	4.04 ± 0.29 ***

HBM, Health Belief Model; ** significant at *p* < 0.01 between respective groups (independent samples *t*-test, equal variance not assumed), and within groups, except “*perceived benefit*” (paired samples *t*-test, equal variance not assumed); *** difference significant at *p* < 0.001 between respective group (independent-samples *t*-test, equal variance not assumed) and within group (paired samples *t*-test, equal variance not assumed); ^a^ significant at *p* < 0.001 within group only (paired sample *t*-test, equal variance not assumed).

**Table 4:** (**A**) Consumption index and diet diversity scores for mother and children from two pulse-growing rural communities of Halaba, Ethiopia. (**B**) Group intakes of energy and selected nutrients from single-day weighed food records in a subsample of mothers and their children from two pulse-growing rural communities of Halaba, south Ethiopia.

	Median (Interquartile Range)			
	Control		Intervention	
**(A)**				
	Pre (*n* = 180)	Post (*n* = 180)	Pre (*n* = 183)	Post (*n* = 183)
Mothers Consumption index				
Any animal product	1.3 (1–1.8)	1.3 (1–1.8)	1.3 (1–1.5)	1.5 (1–1.8) ^c^
Any fruits or vegetables	2.5 (2–3) ***	2.5 (2–3)	2.5 (2.5–3)	2.5 (2.3–3)
Any pulse ^a^	2 (1.5–2.3) ***	2 (1.8–2.8) ^c^	2.3 (1.8–2.5)	2.3 (2–2.8) ^c^
DDS	4 (3–4) ***	3 (3–4) ^c^	3 (3–4)	4 (3–4) ^c^
Children (6–59 mos.) ^b^ Consumption index				
Any animal product	1.3 (1–1.8)	1.5 (1–1.8)	1.3 (1.3–1.8)	1.5 (1.3–2) ^c^
Any fruits or vegetables	2.5 (1.5–3) ***	2.5 (2–3)	3 (2.5–3)	3 (2.5–3)
Any pulse ^a^	1.8 (1.5–2.3) ***	2 (1.5–2.5) ^c^	2.3 (1.8–2.6)	2.3 (2–2.8) ^c^
DDS	3 (2.3–4)	3 (3–4) ^c^	3 (3–4)	4 (3–4) ^c^
**(B)**				
	Pre (*n* = 63)	Post (*n* = 63)	Pre (*n* = 59)	Post (*n* = 59)
Mothers				
Energy (kcal) ^a^	1410 (1128–1929)	1615 (1314–1892)	1669 (1431–1959) ^b^	1711 (1469–2011)
RNI (% RNI)	2700 (52)	2700 (60)	2700 (62)	2700 (63.4)
Protein (g)	53 (41–63)	57 (46–67)	61 (53–69) ^b^	57 (46–66)
RNI (% RNI)	42 (126)	42 (136)	42 (145)	42 (136)
Iron (mg)	60 (42–73)	70 (50–111) **	66 (51–87)	74 (50–169) *
RNI (% RNI)	58.8 (102)	58.8 (119)	58.8 (112)	58.8 (126)
Zinc (mg)	13 (9–15)	13 (10–16)	14 (11–17)	13 (10–16)
RNI (% RNI)	9.8 (133)	9.8 (133)	9.8 (143)	9.8 (133)
Calcium (mg)	758 (514–1140)	841 (635–1069)	983 (588–1178)	709 (564–988) * ^c^
RNI (% RNI)	1000 (76)	1000 (84)	1000 (98)	1000 (71)
Children (6–59 mos.)				
Energy (kcal)	315 (184–545)	569 (423–790) ***	426 (256–614)	608 (488–783) ***
RNI	-	-	-	-
Protein (g)	12 (7–19)	18 (14–27) ***	16 (11–23)	20 (16–25) **
RNI	10–17	10–17	10–17	10–17
Iron (mg)	11 (6–26)	23 (16–46) ***	18 (12–30) ^b^	29 (21–60) *** ^c^
RNI	18.6–12.6	18.6–12.6	18.6–12.6	18.6–12.6
Zinc (mg)	2.8 (1.5–4.6)	4.3 (3.3–6.1) ***	3.4 (2.3, 5)	4.9 (3.3–6.2) ***
RNI	8.4–9.6	8.4–9.6	8.4–9.6	8.4–9.6
Calcium (mg)	167 (82–338)	298 (186–404) **	230 (141–364)	330 (215–439) *
RNI	400–600	400–600	400–600	400–600

(**A**) ^a^ Includes lentils, peas, kidney beans and broad beans. ^b^ *n* = 113 in control, *n* = 126 in intervention, excluding children <6 months; Consumption index: 1 = least frequent (≤ 2 per month), 2 = 1–2 times per week, 3 = 3–6 times per week, 4 = most frequent (≥1 per day); *** significant at *p* < 0.001 (*Mann–Whitney U*, 2-tailed) between groups. ^c^ estimates significantly different at *p* < 0.05 (*Wilcoxon,* 2-tailed) within groups (differences in DDS and pulse consumption index of mothers among the control group were very significant at *p* < 0.001). (**B**) ^a^ The daily recommendation of 2700 kcal chosen from FAO/WHO/UNU joint expert consultation report for human energy requirement [30] and assumes an average weight of 50 kg and a physical activity level of 2.2 (the median for vigorous or vigorously active lifestyle) for rural mothers; RNI, recommended nutrient intake: assumes 5% bioavailability for iron and low bioavailability for zinc, based on WHO/FAO recommendations [31]. RNI values for protein are based on WHO/FAO/UNU recommendations [32] and were adjusted by average weight for the mothers; ***, **, * significant within groups at *p* < 0.001, *p* < 0.01, and *p* < 0.05, respectively *(Wilcoxon test, non-parametric)*; ^b^ significant at *p* < 0.05 between groups at baseline *(Mann–Whitney U test)*; ^c^ significant at *p* < 0.05 between groups at endline *(Mann–Whitney U test).*

**Table 5:** Results from anthropometric measurements and associated indices for mothers and children from two pulse-growing rural communities of Halaba, Ethiopia.

	Mean ± SD or %			
	Control		Intervention	
	Pre (*n* = 161)	Post (*n* = 161)	Pre (*n* = 165)	Post (*n* = 165)
Mothers				
Height (cm)	158 ± 6	158 ± 6	156 ± 5	156 ± 5
MUAC (cm)	25 ± 3	25 ± 3	24 ± 2	24 ± 2
Weight (kg) ^a^	52 ± 7	52 ± 7	48.7 ± 5.4	49 ± 5.3
BMI (kgm^−2^) ^a^	21 ± 2.4	20.8 ± 2.3	19.9 ± 1.8	20 ± 2
Underweight, BMI < 18.5	25/136 (18.4)	17/136 (12.5)	32/137 (23.4)	34/137 (24.8)
Normal, BMI 18.5–24.99	104/136 (76.5)	113/136 (83.1)	105/137 (76.6)	101/137 (73.7)
Overweight, BMI 25–29.99	7/136 (5.1)	6/136 (4.4)	0/137 (0)	2/137 (1.5)
Children (0–59 mos.) ^b^				
Height-for-age Z-score	−1.87 ± 1.7 * ^c^	−2.24 ± 1.3	−2.24 ± 1.4	−2.53 ± 1.16 * ^d^
Weight-for-height Z-score	−0.47 ± 1.3 *	−0.29 ± 1.04	−0.55 ± 1.22	−0.47 ± 1
Weight-for-age Z-score	−1.43 ± 1.48	−1.42 ± 1.17	−1.69 ± 1.35	−1.71 ± 1.1^d^
BMI-for-age Z-score	−0.35 ± 1.23 *	0.03 ± 1.03	−0.42 ± 1.22	−0.13 ± 1.02 *
Prevalence of stunting	75/164 (45.7) * ^c^	89/164 (54.3)	96/162 (59.3)	110/162 (67.9) ^d^ *
Prevalence of wasting	18/164 (11) *	6/164 (3.7)	18/162 (11.1)	8/162 (4.9) *
Prevalence of underweight	50/164 (30.5)	50/164 (30.5)	68/162 (42)	59/162 (36.4)

^a^ Excludes pregnant or pregnant and lactating or those who had babies with in the last two months prior to the anthropometric measurement (*n* = 136 in control, *n* = 137 in intervention); ^b^ *n* = 164 in control, *n* = 162 in intervention; * significant at *p* < 0.05 within respective groups (*paired sample t-test for the Z-scores, and McNemar test for prevalence estimates*); ^c^ significant at *p* < 0.05 between respective groups (*independent sample t-test for the mean Z-scores and Pearson’s chi-squared test* for prevalence estimates); ^d^ significant at *p* < 0.05 between groups (*independent sample t-test for the mean Z-scores and Pearson’s chi-squared test* for prevalence estimates).

## Data Availability

The original contributions presented in the study are included in the article and Supplementary Material, further inquiries can be directed to the corresponding author.

## Supplementary Materials

The following supporting information can be downloaded at: https://www.mdpi.com/article/10.3390/children11111400/s1, Table S1: Summary of nutrition education topics, content highlights and key messages, offered in rural communities of Halaba, south Ethiopia, 2013–2014.
