# Supplementary material for: A Nutrition Education Intervention Positively Affects the Diet–Health-Related Practices and Nutritional Status of Mothers and Children in a Pulse-Growing Community in Halaba, South Ethiopia
Source: Children (Basel). 2024 Nov 19;11(11):1400. doi: 10.3390/children11111400 (PMC11592757; doi:10.3390/children11111400)
Supplement: Supplementary file 1 [file children-11-01400-s001.zip › children-3305072-supplementary.pdf]

Supplementary Table S1. Summary of nutrition education topics, content highlights and key messages, offered in rural communities of Halaba, south Ethiopia, 2013–2014.

Session	Topics	Content highlights	Key nutrition messages
1	Tips on nutrition during pregnancy, lactation early childhood.	• Importance of healthy eating before and during pregnancy and lactation for the health of both mother and child.<br>• Understanding the increased nutritional needs of mothers and growing children and hence prioritizing them during intra-household food distribution.	• Prioritise the nutrition of pregnant & lactating mothers and children.<br>• Mothers and children should be given priority in the intra-household food distribution.
2	Food groups & diversifying diets	• Understanding the concept of food groups and diversifying diet.	• A healthy meal/diet is one that includes variety of foods from the different food groups.
3	Improving protein quality of cereal-based foods with pulses	• Important contribution of pulses as good sources of protein and micronutrient.<br>• How protein quality of cereals can be improved by combining them with pulses crops.	• Pulses are great sources of protein and micronutrients needed for the building and healthy functioning of our body.<br>• Combining foods from cereals with pulses (e.g. kidney beans) makes a good quality protein (complete protein).
4	Consumption of fruits & vegetables	• Understanding the importance of including fruits and vegetables in the diet.	• Fruits and vegetables are excellent sources of nutrients that help our body fight off disease/sickness.
5	Benefits of pulses	• Benefits of pulse agriculture in improving soil fertility, saving the money for other household needs which would otherwise be spent for purchase of artificial fertilizer.<br>• Source of cash income through selling of pulses in markets as they are high-value crops.	• Pulses are nutritious, good for health and contribute to healthy environment by making their own natural fertilizer.<br>• Pulses are high-value crops that can generate cash-income for households.
6	Sanitation/hygiene	• Basics of personal and environmental hygiene,<br>• Hygiene during child feeding (breast feeding and complementary feeding) practices;	• Maintain a healthy environment for children; keep your homestead clean by practicing proper liquid and solid waste disposal<br>• Handle food hygienically by practicing handwashing as often as possible.
